# Gut Microbiome Signatures Are Biomarkers for Cognitive Impairment in Patients With Ischemic Stroke

**DOI:** 10.3389/fnagi.2020.511562

**Published:** 2020-10-23

**Authors:** Yi Ling, Tianyu Gong, Junmei Zhang, Qilu Gu, Xinxin Gao, Xiongpeng Weng, Jiaming Liu, Jing Sun

**Affiliations:** ^1^Department of Neurology, The Second Affiliated Hospital and Yuying Children’s Hospital of Wenzhou Medical University, Wenzhou, China; ^2^Department of Preventive Medicine, School of Public Health and Management, Wenzhou Medical University, Wenzhou, China

**Keywords:** biomarkers, ischemic stroke, *Enterobacteriaceae*, cognitive impairment, gut microbiome

## Abstract

Post-stroke cognitive impairment (PSCI) is a common neuropsychiatric complication of stroke. Mounting evidence has demonstrated a connection between gut microbiota (GM) and neuropsychiatric disease. Our previous study revealed the changes in the GM in a mouse model of vascular dementia. However, the characteristic GM of PSCI remains unclear. This study aimed to characterize the GM of PSCI and explored the potential of GM as PSCI biomarkers. A total of 93 patients with ischemic stroke were enrolled in this study. The patients were divided into two groups according to their MoCA scores 3 months after stroke onset. Clinical data and biological variables were recorded. GM composition was analyzed using 16S ribosomal RNA sequencing, and the characteristic GM was identified by linear discriminant analysis Effect Size (Lefse). Our results showed that *Proteobacteria* was highly increased in the PSCI group compared with the post-stroke non-cognitive impairment (PSNCI) group, the similar alterations were also observed at the class, order, family, and genus levels of *Proteobacteria*. After age adjustments, the abundance of *Firmicutes*, and its members, including *Clostridia*, *Clostridiales*, *Lachnospiraceae*, and *Lachnospiraceae_other*, were significantly decreased in the age-matched PSCI group compared with the PSNCI group. Besides, the GM was closely associated with MoCA scores and the risk factors for PSCI, including higher baseline National Institute of Health Stroke Scale score, higher homocysteine (Hcy) level, higher prevalence of stroke recurrence, leukoaraiosis, and brain atrophy. The KEGG results showed the enriched module for folding, sorting and degradation (chaperones and folding catalysts) and the decreased modules related to metabolisms of cofactors and vitamins, amino acid, and lipid in PSCI patients. A significant correlation was observed between PSCI and the abundance of *Enterobacteriaceae* after adjustments (*P* = 0.035). Moreover, the receiver operating characteristic (ROC) models based on the characteristic GM and *Enterobacteriaceae* could distinguish PSCI patients from PSNCI patients [area under the curve (AUC) = 0.840, 0.629, respectively]. Our findings demonstrated that the characteristic GM, especially *Enterobacteriaceae*, might have the ability to predict PSCI in post-stroke patients, which are expected to be used as clinical biomarkers of PSCI.

## Introduction

Ischemic stroke is a major risk factor for cognitive impairment (Vijayan and Reddy, 2016b). The occurrence of cognitive impairment after stroke may be the result of vascular cognitive impairment or Alzheimer’s disease (AD) promoted by stroke, or both (Sun et al., 2014). Zekry et al. (2003) revealed that the infarcts in strategic regions are critical for the pathogenesis of cognitive impairment after stroke. Besides, stroke and cognitive impairment also share similar risk factors such as hypertension and diabetes mellitus, which contribute to cognitive impairment after stroke (Sun et al., 2014). Therefore, ischemic stroke is closely correlated with cognitive impairment. Post-stroke cognitive impairment (PSCI) is a common complication of stroke. In China, the prevalence of cognitive impairment 3 months after stroke ranges from 18 to 41.8% (Tang et al., 2006; Tu et al., 2014). PSCI is associated with poor clinical outcomes such as increased hospitalization, disability, and burden of care (Crichton et al., 2016), and functional impairment is more significant in stroke survivors with cognitive impairment. Previous studies have focused on the demographic, psychological, and biological variables influencing PSCI (Arba et al., 2017; Levine et al., 2018). However, the pathogenesis of PSCI remains unclear. Given that there is a prodromal period after stroke onset of 3 months or more before the development of PSCI (Ballard et al., 2003), it is of considerable significance to identify useful PSCI biomarkers.

Gut microbiota (GM) dysbiosis in neuropsychiatric disorders has been observed in human and animal studies. Recent studies showed that fecal microbial diversity and composition were significantly different between AD patients and healthy controls (Zhuang et al., 2018). The GM of AD patients was characterized by a higher abundance of bacteria inducing proinflammatory states, and a lower abundance of bacteria able to synthesize short-chain fatty acids (SCFAs) (Haran et al., 2019). The animal study confirmed the altered GM in a mouse model of AD, which was characterized by increased abundances of *Verrucomicrobia* and *Proteobacteria*, and decreased levels of *Ruminococcus* and *Butyricicoccus* (Zhang et al., 2017). Moreover, our previous study demonstrated that fecal microbiota transplantation could reduce AD symptoms in the APP/PS1 mouse model (Sun et al., 2019). Besides, patients with schizophrenia exhibited decreased GM diversity and microbial dysbiosis (Xu et al., 2019b), and transplantation of gut bacteria from schizophrenic patients into antibiotic-treated mice caused schizophrenia-like abnormal behaviors (Zhu et al., 2019). Increased abundances of opportunistic pathogens and decreased levels of butyrate-producing bacteria were identified as hallmarks of post-stroke GM dysbiosis (Yin et al., 2015). Animal studies indicated that the GM dysbiosis exacerbated the outcome of stroke, while transplantation of fecal microbiota or normalization of GM dysbiosis by antibiotics improved the outcome (Singh et al., 2016; Chen et al., 2019). Moreover, increasing evidence indicated the close correlation between the GM and cognitive impairment in different diseases (Bajaj et al., 2012; Carlson et al., 2018; Gao et al., 2019; Liu et al., 2019). However, the gut microbial characteristics in PSCI patients remain unclear.

Many studies used animal models to investigate the role of GM in the brain function, such as germ-free mice, and animal models treated with probiotics. For example, the germ-free mice showed impaired social behaviors (Diaz Heijtz et al., 2011; Neufeld et al., 2011), and structural alterations in the amygdala and prefrontal cortical (Stilling et al., 2015; Hoban et al., 2016). The previous study of germ-free animals had indicated that GM regulated neurogenesis, which modulated learning and memory (Ogbonnaya et al., 2015). Administration of probiotics to healthy rats and mice showed the alleviation of anxiety-like and depression-like behaviors (Dinan et al., 2013). Moreover, oral treatment with SCFAs could alleviate the impaired microglial function in germ-free animals, according to the previous study (Erny et al., 2015). Besides, fecal microbiota transplantation could transfer behavioral phenotypes (Collins et al., 2013). However, these studies were based on animal models, whether these findings of animal studies could be generalized to humans remained unclear. Therefore, there is a need to elucidate the relationship between GM and neuropsychiatric diseases in human studies.

In the present study, we aimed to investigate the GM composition in PSCI patients and GM’s association with MoCA scores and risk factors for PSCI. Besides, we further confirmed the characteristic GM of PSCI and its potential as a biomarker for the diagnosis of PSCI.

## Materials and Methods

### Study Patients

Ischemic stroke patients diagnosed and treated in the Second Affiliated Hospital and Yuying Children’s Hospital of Wenzhou Medical University from January to April 2019 were enrolled. The inclusion criteria were as follows: patients aged 40–90 years, ischemic stroke, with infarcts in non-strategic brain regions (including the subcortex, brain stem, and cerebellum). Exclusion criteria included the following: pre-existing dementia history, infarct of strategic regions (hippocampus, thalamus, frontal lobe, cingulate gyrus, angular gyrus, internal capsule, caudate nucleus), recent (within 3 months) use of antibiotics or probiotics, restrictive diet, gastrointestinal surgery, recent infection, psychosis such as schizophrenia or bipolar disease, severe life-threatening illnesses, communication deficits, and pregnancy. The Ethics Committee of the Second Affiliated Hospital of Wenzhou Medical University approved the study protocol, and all patients gave written informed consent.

### Neuropsychological Assessment

Patients were assessed by the Montreal Cognitive Assessment (MoCA) 3 months after stroke onset. The MoCA, characterized by excellent specificity and sensitivity (Zietemann et al., 2018), is currently the most widely used tool to assess cognitive function, and includes visuospatial/executive function, naming, attention, abstraction, language, delayed recall, and orientation. We used the score of the Informant Questionnaire on Cognitive Decline in the Elderly (cut-off value > 4.0) to exclude pre-existing dementia. Patients were identified as PSCI as follows: MoCA score < 26 points for patients with junior school education level or above, MoCA score < 21 points for patients with primary school education level, and MoCA score < 15 points for illiterate patients. The remaining patients were identified as post-stroke non-cognitive impairment (PSNCI).

### Clinical Data Collection

We collected the demographic information, including sex, age, divorce rate, and educational level, physical activity, sleep deprivation, smoking and alcohol status, previous history of stroke, and dietary habit of each patient from an interview, and the height and weight of each patient were obtained to calculate the body mass index (BMI). We determined whether the patients had hypertension, diabetes mellitus, dyslipidemia, and atrial fibrillation by inquiring about the history of previous diseases and measuring patients’ blood pressure, blood glucose, blood lipid, and electrocardiogram, respectively. In addition, patients were examined by brain magnetic resonance imaging (MRI) scans to determine whether there was leukoaraiosis (LA) and brain atrophy, which was performed on a 1.5-T scanner (GE Discovery750, Milwaukee, United States) using standard protocols. We measured serum Hcy level (μmol/L) in each patient using standard enzymatic methods (A15 Random Access Analyzer, Biosystems, Spain). The professional neurologist assessed the National Institute of Health Stroke Scale (NIHSS) score and MoCA score of each patient.

### Sample Collection and Processing

All patients provided fresh stool within 1 week of admission. Stool samples were collected using the MiSeq Reagent kit (PE300 v3) and immediately transferred to the laboratory for repackaging within 15 min. The 200 mg feces samples were placed into a 2 ml sterile centrifuge tube and divided into three parts and labeled, respectively. All specimens were processed within 30 min after collection, and the samples were stored at −80°C. Fecal genomic DNA was extracted from stool samples using a DNA extraction kit (TIANGEN, TIANamp, China), according to the manufacturer’s methods, as described in previous studies (Li et al., 2008; Shkoporov et al., 2018). The fecal samples were lysed in lysis buffer, and we put VAHTS DNA Clean Beads in it, then homogenizing for 3–5 min in a vortex mixer (Qilinbeier Vortex-5), purified with 200 μl 80% ethanol, and eluted with 24 μl of elution buffer. The quantity of extracted genomic DNA was evaluated by 2% agarose gel, and DNA purity and concentration were determined by NanoDrop spectrophotometer (Thermo Fisher Scientific, United States). A260/A280 ratios were also measured to confirm the high-purity of the DNA yield. Then we stored the extracted DNA at −20°C.

The DNA extraction was followed by the amplification of the V3–V4 16S ribosomal RNA gene region, with the forward primer (5′-CCTACGGGNGGCWGCAG-3′) and the reverse primer (5′-GACTACHVGGGTATCTAATCC-3′), as described in the previous study (Bu et al., 2018). The PCR process was as follows: denaturation at 95°C for 30 s, annealing at 55°C for 30 s, extension at 72°C for 45 s, 25 cycles, and a final extension at 72°C for 5 min. Reaction system: 2 × Phanta Max Master Mix 25 μl, DNA template 5 μl, Nextera XT Index Primer 1 2 μl, Nextera XT Index Primer 2 2 μl, ddH_2_O 16 μl. PCR products were validated in a 2% agarose gel for single bands and expected sizes.

### Sequence Processing and Analysis

The DNA libraries were pooled and sequenced on a MiSeq Benchtop Sequencer (Illumina, Singapore, United States). For quality control, the reads without primers were discarded using cutadapt, version 1.11, and the chimeric reads removed. The processed pair-end reads were merged using PandaSeq, version 2.9, with default parameters, to generate representative complete nucleotide sequences. The overlapping areas of the paired-end reads were processed first, and low-quality reads (average *Q* < 20) and those containing ambiguous bases denoted by Ns were deleted. Vsearch was used to cluster high-quality sequences with a similarity cut-off of 0.97. We selected the sequences with the highest abundance in each class as the representative. The representative sequences were annotated (down to the genus) using the RDP classifier, version 2.12 (Whelan and Surette, 2017), and sequences which could not be assigned to any specific classification level were labeled as “unclassified.” QIIME was used to remove the Operational Taxonomy Units (OTUs) with only one sequence in all samples.

### Bioinformatics and Data Analysis

Bacterial diversity was determined by α-diversity (Shannon’s index and Simpson index) and β-diversity (Principal coordinates analysis, PCoA). The α-diversity indices were analyzed using the R software. A Mann-Whitney *U*-test or Kruskal Wallis H was performed to compare the α-diversity of groups. The β-diversity comparison was performed by analysis of similarities using the Bray-Curtis dissimilarity index. Significant *P*-values associated with microbial clades and functions were identified by linear discriminant analysis Effect Size (Lefse) (Qian et al., 2018). The Lefse analysis used the Kruskal-Wallis test (alpha value of 0.05) and a linear discriminant analysis score > 2 as thresholds. We used Phylogenetic Investigation of Communities by Reconstruction of Unobserved States (PICRUSt) to predict gene contents and metagenomic functional information according to the OTU table (Langille et al., 2013). We used the receiver operating characteristic (ROC) curves and the area under the curve (AUC) to verify the specificity and sensitivity of the characteristic GM in diagnosing PSCI, and investigate whether the characteristic GM could be regarded a biomarker for PSCI.

### Statistical Analysis

Statistical analysis was carried out using GraphPad Prism V.5.0.1 (La Jolla, CA, United States), the R software (V.3.5), Adobe Illustrator CC 2015 (Adobe Systems Incorporated, California, America), and SPSS, V.22 (SPSS, Chicago, United States). Categorical variables were presented as numbers and percentages and compared by chi-squared test. Continuous variables were described as mean and standard deviation or median and interquartile range, depending on the outcome of a Kolmogorov-Smirnov normality test, and compared by Student’s *t*-test or Mann-Whitney test, respectively. Mann-Whitney test was used to determine the significance of the difference between PSCI and PSNCI groups (i.e., PSCI vs. PSNCI; age-matched PSCI vs. PSNCI). We used multivariable logistic regression to determine the risk factors for PSCI and the representative microbiota associated with PSCI after adjustments for age and the risk factors. The probability cut-offs to enter or remove a variable were 0.05 and 0.1, respectively. Spearman rank correlation was used to analyze GM’s correlation with MoCA scores and the risk factors for PSCI. We further selected 29 PSCI patients as a subgroup of younger PSCI with average age similar to the PSNCI group. Randomization was stratified by age.

## Results

### Baseline Characteristics of the Recruited Patients

**FIGURE 1:**
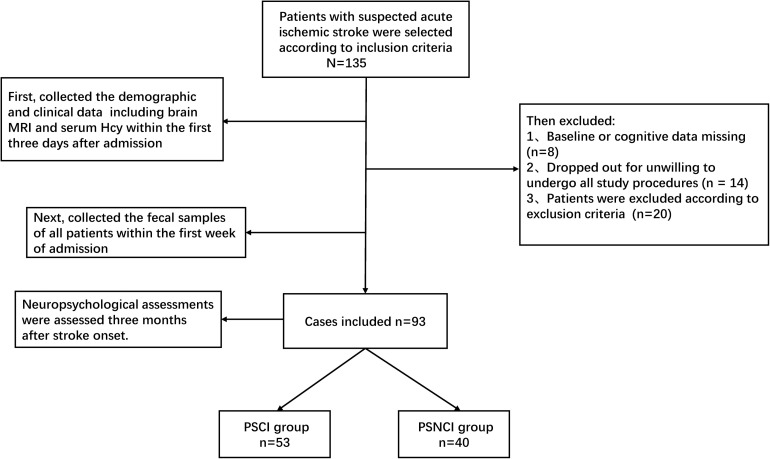
Flowchart of patients included in this study.

At first, a total of 135 stroke patients were enrolled, 14 patients were excluded due to unwillingness to participate in all the study procedures, eight patients were excluded due to missing data, and 20 patients were excluded according to the exclusion criteria, leaving 93 patients that could be analyzed (Figure 1). The patients’ demographic information and MoCA scores in the two groups (53 and 40 patients in the PSCI and PSNCI group, respectively) are summarized in Table 1. There were significant differences in terms of age, NIHSS and MoCA scores, stroke recurrence (not the first stroke), Hcy, LA, and brain atrophy between the two groups (*P* = 0.006, 0.001, < 0.001, < 0.001, < 0.001, < 0.001, < 0.001, respectively). After age-matched, the NIHSS and MoCA scores, stroke recurrence, Hcy, LA, and brain atrophy still exhibited the significant differences between the two groups (Supplementary Table S1). However, no significant difference was found in sex, divorce rate, education level, physical activity, sleep deprivation, diabetes mellitus, hypertension, dyslipidemia, atrial fibrillation, BMI, current smoking and alcohol status, and dietary risks between the two groups.

**TABLE 1:** The significant differences of demographic and clinical parameters between PSCI and PSNCI groups.

Characteristic	PSCI group (*n* = 53)	PSNCI group (*n* = 40)	*P-*value
Male	66.0	67.5	0.882
Age, years	72.2 ± 10.3	66.0 ± 10.8	0.006
Divorce rate	15.1	10.0	0.468
Low education (<high school)	71.7	57.5	0.154
Physical activity (≥90 min/week)	43.4	62.5	0.068
Sleep deprivation (<6 h)	52.8	45.0	0.455
NIHSS score	3 (1–5)	1 (1–2)	0.001
MOCA score	13.7 ± 6.7	22.3 ± 4.2	<0.001
Visuospatial/executive function	2 (1–3)	3 (2–3)	<0.001
Naming	1 (0–2)	2 (1–3)	0.002
Attention	4 (2–5)	6 (5–6)	<0.001
Language	2 (1–2)	3 (2–3)	<0.001
Abstraction	0 (0–1)	1 (0–1)	0.003
Delayed recall	1 (0–3)	4 (3–4)	<0.001
Orientation	3 (2–5)	6 (5–6)	<0.001
Diabetes mellitus	24.5	32.5	0.396
Hypertension	77.4	65.0	0.189
Dyslipidemia	51.9	42.5	0.370
Atrial fibrillation	17.0	7.5	0.177
Stroke recurrence	58.5	20.0	<0.001
BMI (kg/m^2^)	24.7 ± 3.7	25.5 ± 3.3	0.263
Current smokers	17.0	15.0	0.797
Alcohol drinker	41.5	43.6	0.842
Hcy (μmol/L)	13.3 ± 4.7	10.2 ± 2.4	<0.001
Dietary risks			
High fat	67.9	72.5	0.634
Low in fruits	52.8	45.0	0.455
Low in vegetables	41.5	45.0	0.736
LA	96.2	67.5	<0.001
Brain atrophy	67.9	22.5	<0.001

The comparisons of the sub-items of the MoCA score between the two groups were shown in Table 1. PSCI patients had lower scores in all sub-items, including visuospatial/executive function, naming, attention, language, abstraction, delayed recall, and orientation (all *P <* 0.005).

### Multivariable Logistic Regression Analysis of the Risk Factors for PSCI

Multivariable logistic regression was used to evaluate which variables could represent risk factors for PSCI. PSCI was independently associated with higher baseline NIHSS score [OR 1.553, 95% confidence interval (CI) 1.014–2.379, *P* = 0.043], higher Hcy level (OR 1.219, 95% CI 1.013–1.466, *P* = 0.036), higher prevalence of stroke recurrence (OR 4.042, 95% CI 1.293–12.634, *P* = 0.016), brain atrophy (OR 3.663, 95% CI 1.181–11.359, *P* = 0.025), and higher proportion of LA (OR 8.780, 95% CI 1.210–63.729, *P* = 0.032) after adjustment for age (Table 2).

**TABLE 2:** Multivariate logistic regression analysis of risk factor for PSCI after adjustment for age.

Variable	Multivariate			
	B (SE)	OR	95% CI	*P*-value
NIHSS score	0.440 (0.218)	1.553	1.014–2.379	0.043
Stroke recurrence	1.397 (0.581)	4.042	1.293–12.634	0.016
Hcy	0.198 (0.094)	1.219	1.013–1.466	0.036
LA	−2.173 (1.011)	8.780	1.210–63.729	0.032
Brain atrophy	1.298 (0.577)	3.663	1.181–11.359	0.025

### Alterations of GM Composition in PSCI Patients

**FIGURE 2:**
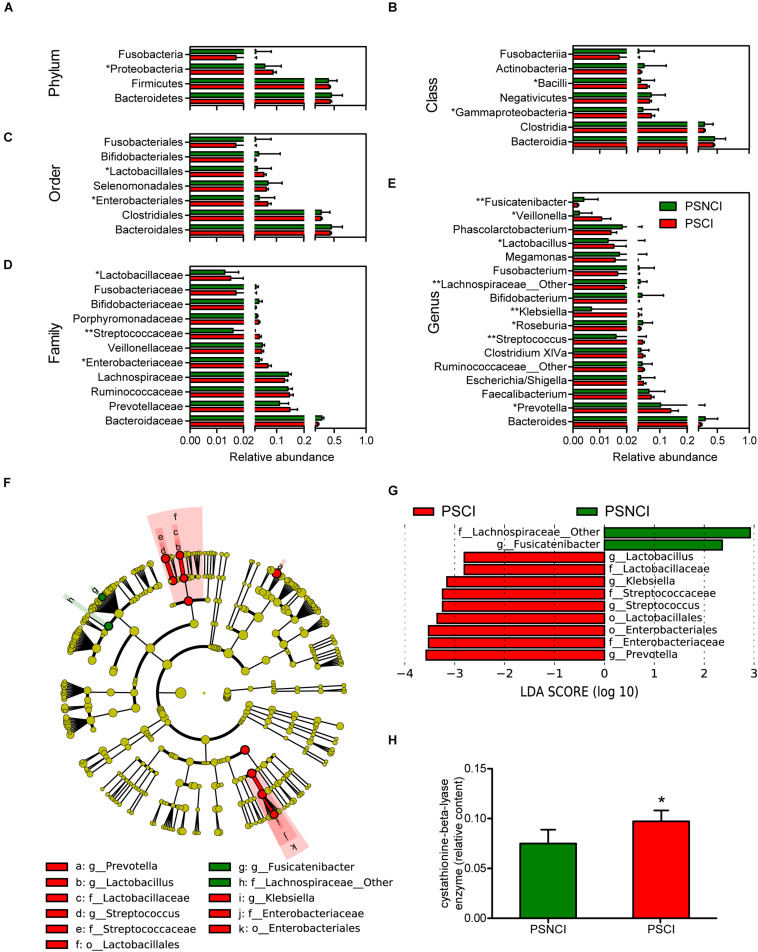
Comparison of the representative taxonomic abundance between post-stroke cognitive impairment (PSCI) and post-stroke non-cognitive impairment (PSNCI) groups. **(A)** Mann-Whitney *U*-test indicated the significant differences in phylum between the two groups and also in their corresponding class **(B)**, order **(C)**, family **(D)**, and genus **(E)**. **(F)** A cladogram of different taxonomic composition between PSCI patients and PSNCI patients. **(G)** linear discriminant analysis scores showed significant bacterial differences between PSCI patients and PSNCI patients. **(H)** Compare the functional Kyoto Encyclopedia of Genes and Genomes orthology of gut microbiota in PSCI and PSNCI groups. Mann-Whitney *U*-test indicated the significant differences between the two groups. o, order; f, family; g, genus. *P < 0.05, **P < 0.01.

Analysis of the 16S ribosomal RNA sequencing gave a total of 197,660 OTUs, classified into 14 phyla, 28 classes, 50 orders, 97 families, and 243 genera. As shown in Supplementary Figure S1C, although no significant difference in gut bacterial communities between the PSCI and PSNCI groups was evident from the PCoA scatterplot, the relative abundances of some gut microbial taxa were significantly different between the two groups. At the phylum level, patients with PSCI had a significantly higher content of *Proteobacteria* (8.7 vs. 5.7%, *P* = 0.016, Figure 2A). At the class level, patients with PSCI had higher contents of *Gammaproteobacteria* (6.9 vs. 3.8%, *P* = 0.017, Figure 2B) and *Bacilli* (5.3 vs. 3.0%, *P* = 0.012, Figure 2B). At the order level, PSCI was associated with significantly higher abundances of *Enterobacteriales* (6.8 vs. 3.7%, *P* = 0.013, Figure 2C) and *Lactobacillales* (5.3 vs. 3.0%, *P* = 0.011, Figure 2C). At the family level, patients with PSCI had higher contents of *Enterobacteriaceae* (6.8 vs. 3.7%, *P* = 0.013, Figure 2D), *Streptococcaceae* (3.6 vs. 1.6%, *P* = 0.005, Figure 2D), and *Lactobacillaceae* (1.5 vs. 1.3%, *P* = 0.02, Figure 2D). At the genus level, PSCI patients had significantly higher levels of *Streptococcus* (3.6 vs. 1.6%, *P* = 0.005, Figure 2E), *Klebsiella* (2.3 vs. 0.6%, *P* = 0.002, Figure 2E), *Lactobacillus* (1.5 vs. 1.3%, *P* = 0.02, Figure 2E), *Prevotella* (14.0 vs. 10.2%, *P* = 0.01, Figure 2E), and *Veillonella* (1.05 vs. 0.23%, *P* = 0.022, Figure 2E); and lower contents of *Roseburia* (2.7 vs. 3.7%, *P* = 0.033, Figure 2E), *f_Lachnospiraceae_other* (1.9 vs. 2.8%, *P* = 0.008, Figure 2E) and *Fusicatenibacter* (0.17 vs. 0.40%, *P* = 0.0018, Figure 2E). However, no significant difference was found between the PSCI and PSNCI groups in fecal microbiota α-diversity (Supplementary Figures S1A,B). Furthermore, as shown in Figure 2H, the relative content of cystathionine-beta-lyase was significantly higher in the PSCI group compared with the PSNCI group (*P* = 0.011).

After the groups were age-matched, although no significant difference in gut bacterial communities among the PSCI, PSNCI, and age-matched PSCI groups was evident from the PCoA scatterplot, the relative abundances of some gut microbial taxa were significantly different between age-matched PSCI and PSNCI groups (Supplementary Figure S3C). As shown in Supplementary Figures S4A–E, at the phylum level, age-matched PSCI patients had a significantly higher content of *Proteobacteria* (age-matched PSCI vs. PSNCI: 10.8 vs. 5.7%, *P* = 0.017), and lower abundance of *Firmicutes* (age-matched PSCI vs. PSNCI: 33.2 vs. 40.7%, *P* = 0.027). The similar alterations were also observed at the class, order, family, and genus levels of *Proteobacteria* and *Firmicutes*, including *Gammaproteobacteria* (age-matched PSCI vs. PSNCI: 9.1 vs. 3.8%, *P* = 0.040), *Clostridia* (age-matched PSCI vs. PSNCI: 21.8 vs. 29.8%, *P* = 0.056), *Enterobacteriales* (age-matched PSCI vs. PSNCI: 9.1 vs. 3.7%, *P* = 0.020), *Clostridiales* (age-matched PSCI vs. PSNCI: 21.8 vs. 29.8%, *P* = 0.056), *Enterobacteriaceae* (age-matched PSCI vs. PSNCI: 9.1 vs. 3.7%, *P* = 0.020), *Klebsiella* (age-matched PSCI vs. PSNCI: 3.2 vs. 0.7%, *P* = 0.031), and *Lachnospiraceae_other* (age-matched PSCI vs. PSNCI: 1.6 vs. 3.0%, *P* = 0.009). Besides, PSCI patients were also associated with a significantly higher abundance of *Prevotella* (age-matched PSCI vs. PSNCI: 19.6 vs. 10.2%, *P* = 0.021). However, no significant difference was found among the three groups in fecal microbiota α-diversity (Supplementary Figures S3A,B).

We further confirmed the characteristic GM using Lefse analysis. Of note, PSCI was associated with increased abundances of *Enterobacteriaceae*, *Klebsiella* of *Enterobacteriales*, and *Lactobacillaceae, Streptococcaceae*, *Streptococcus*, *Lactobacillus* of *Lactobacillales* and *Prevotella*, and decreased abundances of *Fusicatenibacter* and *f_Lachnospiraceae_other* (Figures 2F,G). After being adjusted for age, the age-matched PSCI and PSNCI groups showed significant differences in phylum *Proteobacteria* and *Firmicutes*. The abundances of *Gammaproteobacteria*, *Enterobacteriales*, *Enterobacteriaceae*, *Klebsiella*, and *Prevotella* were significantly higher, the proportions of *Clostridia*, *Clostridiales*, *Lachnospiraceae*, and *Lachnospiraceae_other* were lower in the age-matched PSCI group compared with PSNCI group (Supplementary Figures S4F,G).

The results showed that there was no significant difference in sex between the two groups (PSCI-male, *n* = 35, 66%; PSNCI-male, *n* = 27, 67.5%, *P* = 0.882). According to sex, stroke patients were divided into female and male subjects. The PCoA showed no significant difference in sex between the two groups (Supplementary Figure S5). Moreover, there was no significant difference in the relative abundance of the characteristic gut microbiome between the two groups (Supplementary Figure S6). Therefore, sex may have little effect on gut microbiota composition in this study.

### Predicted Function Analysis of Microbiome

We evaluated the functional differences in the microbiome of PSCI vs. PSNCI. As shown in Supplementary Table S2, the enriched orthologs in PSCI patients were folding, sorting and degradation (chaperones and folding catalysts), genetic information processing (protein folding and associated processing, transcription related proteins), energy metabolism (nitrogen metabolism, sulfur metabolism), metabolism (glycan biosynthesis and metabolism, nucleotide metabolism), enzyme families (protein kinases), carbohydrate metabolism (propanoate metabolism). In contrast, the increased pathways in PSNCI patients were metabolism of cofactors and vitamins (porphyrin and chlorophyll metabolism, pantothenate and CoA biosynthesis, nicotinate and nicotinamide metabolism, thiamine metabolism), amino acid metabolism (phenylalanine, tyrosine and tryptophan biosynthesis, arginine and proline metabolism, histidine metabolism, alanine, aspartate and glutamate metabolism, valine, leucine and isoleucine biosynthesis, valine, leucine, and isoleucine degradation), carbohydrate metabolism, lipid metabolism (primary bile acid biosynthesis, secondary bile acid biosynthesis, linoleic acid metabolism).

### Correlation Between GM Composition and MoCA Score and Its Sub-variables

**FIGURE 3:**
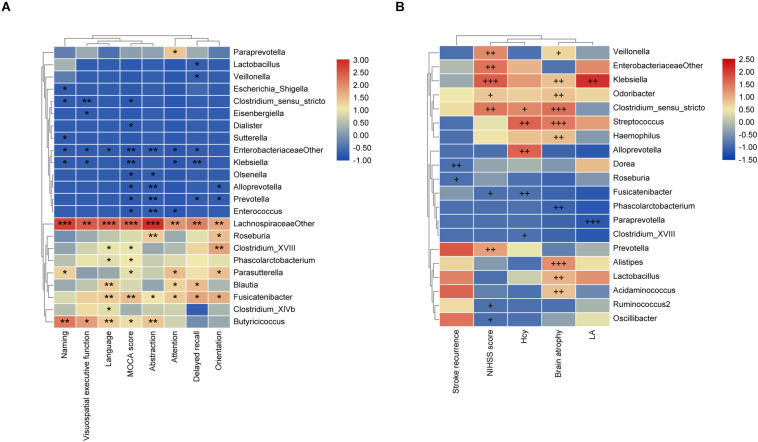
The associations of gut microbiota (GM) with MoCA scores and the risk factors for PSCI. **(A)** Heatmap of spearman rank correlation analysis between GM and MoCA scores and its sub-variables. **(B)** Heatmap of spearman rank correlation analysis between GM and the risk factors for PSCI. Red means positive correlation and blue means negative correlation. *P < 0.05, **P < 0.01, ***P < 0.001, ^+^*P* < 0.1, ^++^*P* < 0.05, ^+++^*P* < 0.01.

The Spearman rank correlation was used to confirm the correlation between MoCA scores and the GM at the genus level. As shown in Figure 3A, *f_Lachnospiraceae_other* (*P* < 0.001), *Fusicatenibacter* (*P* < 0.01), *Parasutterella*, *Phascolarctobacterium*, *Clostridium_*XVIII, and *Butyricicoccus* (*P* < 0.05) were positively associated with the MoCA score, while *Klebsiella*, *Enterobacteriaceae_other* (*P* < 0.01), *Clostridium_sensu_stricto*, *Olsenella*, *Prevotella*, *Dialister*, *Enterococcus*, and *Alloprevotella* (*P* < 0.05) showed negative correlation. Moreover, we further investigated the correlation between gut bacteria and the MoCA sub-items. As shown in Figure 3A, *Fusicatenibacter* was found to be positively associated with delayed recall, orientation, attention, abstraction (*P* < 0.05), and language (*P* < 0.01). *f_Lachnospiraceae_other* was positively correlated with naming, language, abstraction (*P* < 0.001), attention, visuospatial/executive function, delayed recall, and orientation (*P* < 0.01). In addition, *Klebsiella* was negatively associated with delayed recall (*P* < 0.01), attention, visuospatial/executive function, and naming (*P* < 0.05). *Prevotella* was negatively correlated with delayed recall, orientation (*P* < 0.05), and abstraction (*P* < 0.01). *Escherichia/Shigella* was negatively associated with naming (*P* < 0.05).

### Association of GM With Risk Factors for PSCI and PSCI

As shown in Figure 3B, the stroke recurrence was negatively associated with *Roseburia* (*P* < 0.1) and *Dorea* (*P* < 0.05). LA was positively associated with *Klebsiella* (*P* < 0.05), while negatively associated with *Paraprevotella* (*P* < 0.01). Brain atrophy was positively correlated with *Alistipes*, *Streptococcus*, *Clostridium_sensu_stricto* (*P <* 0.01), *Lactobacillus*, *Klebsiella*, *Odoribacter*, *Acidaminococcus*, and *Haemophilus* (*P <* 0.05), but negatively correlated with *Phascolarctobacterium* (*P <* 0.05). The NIHSS score was positively associated with *Klebsiella* (*P* < 0.01), *Veillonella*, *Clostridium_sensu_stricto*, *Enterobacteriaceae_other*, and *Prevotella* (*P* < 0.05), while negatively associated with *Fusicatenibacter* (*P* < 0.1). Moreover, the Hcy level was positively correlated with *Alloprevotella* and *Streptococcus* (*P* < 0.05) but negatively correlated with *Fusicatenibacter* (*P* < 0.05).

In the multivariable logistic regression models (Supplementary Table S3), there was no significant association between the representative microbiota and PSCI in conditions of unadjusted and adjusted for age in models 1 and 2, respectively. However, we observed a significant correlation between PSCI and the abundance of *Enterobacteriaceae* after adjustment for age and risk factors for PSCI, including NIHSS score, stroke recurrence, Hcy, LA, and brain atrophy (*P* = 0.035). Moreover, the higher abundance of *Enterobacteriaceae* represented a closer association with PSCI (*P* = 0.010, OR = 59.721).

### Gut Biomarkers for PSCI

As shown in Supplementary Figure S2, the model based on the Lefse results after being age-matched, which represented the characteristic GM of PSCI, could effectively distinguish PSCI from PSNCI (AUCPS_CI–PSNCI_ = 0.840, 95% CI: 0.760–0.920, *P* < 0.001; AUC _age–matched PSCI–PSNCI_ = 0.858, 95% CI: 0.773–0.944, *P* < 0.001). The model based on the relative abundance of *Enterobacteriaceae* also showed the differentiating effect for PSCI (AUCPS_CI–PSNCI_ = 0.629, 95% CI: 0.510–0.747, *P* = 0.038; AUC_age–matched PSCI–PSNCI_ = 0.658, 95% CI: 0.524–0.792, *P* = 0.029). These results indicated that GM might contain valuable PSCI biomarkers.

## Discussion

In this study, we characterized the GM composition of PSCI patients. Although GM’s bacterial diversity in PSCI patients was similar to that of PSNCI patients, the microbial composition was distinct between the two groups. The abundance of *Proteobacteria* was highly increased in the PSCI group compared with the PSNCI group. Similar alterations were also observed at the class, order, family, and genus levels of *Proteobacteria*. After age adjustments, the abundance of *Firmicutes*, and its members, including *Clostridia*, *Clostridiales*, *Lachnospiraceae*, and *Lachnospiraceae_other*, were significantly decreased in the age-matched PSCI group compared with the PSNCI group. Moreover, we found GM’s close associations with MoCA scores and risk factors for PSCI, including NIHSS score, Hcy, stroke recurrence, LA, and brain atrophy. The abundance of *Enterobacteriaceae* showed a significant correlation with PSCI after adjustments for age and risk factors. Besides, the ROC model, which was based on the characteristic GM, could effectively distinguish PSCI patients from PSNCI patients. In particular, *Enterobacteriaceae* also showed the differentiating ability for PSCI. These results indicated that the GM might provide novel microbiome-related biomarkers for PSCI.

In this study, there were significant differences in terms of NIHSS score, stroke recurrence, Hcy, LA, and brain atrophy between the PSCI and PSNCI groups, and we also observed the associations of these risk factors with GM. Previous studies have revealed that the incidence of post-event dementia was positively correlated with stroke severity (Pendlebury et al., 2019), and GM dysbiosis was positively correlated with NIHSS scores in stroke patients (Xia et al., 2019). Besides, stroke recurrence was a significant contributor to cognitive impairment through its association with white matter hyperintensities (WMH) (Georgakis et al., 2019). In this study, PSCI patients contained a higher abundance of cystathionine beta-lyase, which was involved in the anabolism process of Hcy (Reveal and Paietta, 2013). Many bacteria, yeast, and plants contain the enzyme. Therefore, the changes in serum homocysteine in the PSCI group may be caused by many factors. Hcy levels were positively associated with the risk of cognitive impairment via upregulated pro-inflammatory cytokines, causing endothelial damage and having direct neurotoxic properties (Fang et al., 2014; Di Meco et al., 2018). Previous studies also reported that Hcy levels were associated with increased risk of severe deep and periventricular white matter lesions, contributing to poor cognitive performance (Vermeer et al., 2002), and the strong associations between increased Hcy levels and cognitive decline in patients with AD and Parkinson’s disease had been confirmed (Di Meco et al., 2018; Murray and Jadavji, 2019). Some researchers hypothesized that the Hcy/lipopolysaccharide (LPS) might mediate pyroptosis in the obese adipocytes due to the GM imbalance (Laha et al., 2018), and the altered microbiome in OSAHS patients was associated with Hcy (Ko et al., 2019). Besides, LA also contributed to cognitive deterioration by triggering the release of inflammatory factors (Kaffashian et al., 2016; Hainsworth et al., 2017). An earlier study had shown that generalized brain and hippocampal atrophy contributed to cognitive decline and specifically to memory deficits, through substantial neuronal loss (Fein et al., 2000). Many studies had indicated the alterations of GM in diseases associated with brain tissue atrophy, including AD (Liu et al., 2019) and multiple system atrophy (Wan et al., 2019). Our results supported the evidence from epidemiological studies that identified multiple risk factors for PSCI, including NIHSS score, Hcy, stroke recurrence, LA, and brain atrophy.

Decreased bacterial diversity is observed in various diseases, such as metabolic syndrome (Dabke et al., 2019) and neurodegenerative diseases (Zhang et al., 2017; Zhuang et al., 2018). Gut bacterial diversity is affected by factors such as lifestyle, age, metabolic diseases, and antibiotics (Gulden, 2018; Tengeler et al., 2018). A growing body of evidence has demonstrated that psychotropic drugs could affect the GM profile. Atypical antipsychotics induced a decrease in GM’s diversity and a significant increase in *Lachnospiraceae* abundance and a decrease in *Akkermansia* level in patients with bipolar disease (Flowers et al., 2017). In this study, we had already excluded the patients who had psychosis, such as schizophrenia or bipolar disease. Moreover, we also did not use psychotropic drugs to treat stroke patients during hospitalization. Therefore, we could eliminate the effects of psychotropic drugs on GM. Diet is one of the critical factors in regulating the GM profile. Different diets have different effects on the composition of GM. For example, the administration of a high-fat diet resulted in a decrease in *Bacteroidetes* and a significant increase in the abundance of *Proteobacteria* and *Firmicute* (Hildebrandt et al., 2009). In this study, we classified the diet as high fat, low in fruits, and low in vegetables. The results showed that there were no significant differences in diet between the two groups. Thus, the dietary effects on GM could be avoided. In this study, no significant difference in bacterial diversity was found between the two groups. The similarity in lifestyle between the two groups and the fact that both groups were composed of stroke patients might explain this result.

According to a previous study, age is the confounding factor that may influence the GM composition. The age-related alterations in the GM composition include an increase of *Proteobacteria*, a decrease of the *Firmicutes* to *Bacteroides*, and a reduction of microbiota diversity (Vaiserman et al., 2017). The changes of GM may be associated with inflammation and endotoxin tolerance during the acute phase of stroke and myocardial infarction (Hernandez-Jimenez et al., 2017; Kowalska et al., 2018; Krishnan and Lawrence, 2019). In this study, the increased abundances of *Klebsiella*, *Enterobacteriaceae*, *Enterobacteriales*, *Gammaproteobacteria* of phylum *Proteobacteria*, and *Prevotella* were still found in age-matched PSCI patients compared with PSNCI patients. The previous study had indicated that the enrichment of *Proteobacteria* in the gut reflected dysbiosis of gut microbial community structure and risk of diseases (Shin et al., 2015). Moreover, the increased abundances of *Proteobacteria*, *Gammaproteobacteria*, *Enterobacteriales*, and *Enterobacteriaceae* could lead to the release of proinflammatory cytokine (Dinh et al., 2015; Shin et al., 2015; Sovran et al., 2018), and the proportions of these GM were negatively associated with cognitive function (Liu et al., 2019). The enriched network of taxa containing *Gammaproteobacteria* and *Enterobacteriales* was also observed in colorectal cancer (Peters et al., 2016) and AD patients (Liu et al., 2019), which was consistent with our study. A previous study on liver transplantation reported that the increased abundance of *Klebsiella* was associated with poor cognitive performance (Bajaj et al., 2017), which was in agreement with our results. Administration of *Lactobacillus* improved cognitive functions impaired by chronic restraint stress (Liang et al., 2015) and major depression (Rudzki et al., 2019). However, our results showed that patients with PSCI had more abundance of *Lactobacillus*. Thus, evidence from reports indicated that these gut bacteria might be closely related to PSCI.

We also found a significantly lower abundance of *Firmicutes*, and its members, including *Clostridia*, *Clostridiales*, *Lachnospiraceae*, and *Lachnospiraceae_other*, in age-matched PSCI patients compared with PSNCI patients. According to the previous study, the levels of *Firmicutes* and *Clostridia* were significantly reduced in humans with type 2 diabetes (Larsen et al., 2010). Besides, the decreased abundances of *Firmicutes*, *Clostridia*, *Clostridiales*, and *Lachnospiraceae* had been reported in AD patients (Liu et al., 2019). *Lachnospiraceae* was one of the most abundant known butyrate-producing bacteria in human GM (Hold et al., 2003; Zhang et al., 2019). SCFAs could improve learning and memory function (During et al., 2003), provide neuroprotection and neuroplasticity, and reduce β-amyloid plaques and microglia activation in animal models of AD (Dalile et al., 2019). Chen et al. demonstrated that transplanting fecal bacteria reduced infarct volume and cerebral edemas, and improved cognitive function in rat models of ischemic stroke (Chen et al., 2019). Our previous study also revealed that increasing the content of SCFAs could be a potential treatment for AD via fecal microbiota transplantation (Sun et al., 2019). However, whether inadequate SCFAs-producing bacteria were involved in PSCI still needs to be confirmed by future studies.

Besides, PSCI was associated with several modulations of the Kyoto Encyclopedia of Genes and Genomes (KEGG) pathways. The module for folding, sorting and degradation (chaperones and folding catalysts) progressively enriched in PSCI patients. According to early studies, molecular chaperones and protein-folding catalysts functioned as proinflammatory signals (Henderson and Pockley, 2010), and molecular chaperones were acting as receptors for the major pathogen-associated molecular patterns and LPS (Triantafilou and Triantafilou, 2005), which further induced inflammatory response. However, some evidence indicated that these proteins might have anti-inflammatory actions (Henderson and Pockley, 2010). Besides, the modules related to metabolisms of cofactors and vitamins, amino acid, and lipids were significantly lower in the PSCI patients, which were consistent with the findings in patients with AD (Li et al., 2019). According to the previous study, amino acid reduced inflammation, oxidative stress, and cell death in the gut (Liu et al., 2017). Moreover, the module for secondary bile acids of lipid metabolism could reduce macrophage inflammation and lipoprotein uptake to protect the blood vessels (Pols et al., 2011). These results suggested that multiple and complex communication pathways existed between GM and PSCI.

We also demonstrated the close correlation of GM with MoCA scores and risk factors for PSCI. Notably, we found that *Klebsiella* and *Enterobacteriaceae_other* of family *Enterobacteriaceae* were negatively correlated with the MoCA score, and positively associated with NIHSS score, LA, and brain atrophy. We further confirmed that the abundance of *Enterobacteriaceae* was closely associated with PSCI after adjusting age and risk factors. Furthermore, the ROC model, which was based on the characteristic GM, could effectively distinguish PSCI from PSNCI patients, and *Enterobacteriaceae* also exhibited the differentiating ability for PSCI. According to the previous studies, the abundances of *Enterobacteriaceae* and *Escherichia/Shigella* were increased in patients with AD, these gut bacteria were considered as pro-inflammatory bacteria and induced LPS accumulation, and mediated amyloid aggregation and inflammatory response (Li et al., 2019; Liu et al., 2019). Besides, the increased abundance of *Enterobacteriaceae* was associated with poor prognosis (Xu et al., 2019a). Thus, the increased abundance of *Enterobacteriaceae* might be significantly associated with PSCI.Due to the severity of PSCI, it is urgent to find biomarkers for PSCI diagnosis. Previous studies demonstrated that some microRNAs could achieve expected results in the diagnosis of PSCI (Huang et al., 2016; Wang et al., 2020). Besides, recent studies indicated that the imaging and multiple cellular changes had made significant progress in the diagnosis of neurological disease (Vijayan and Reddy, 2016a; Eyileten et al., 2018; Guo et al., 2018; Vijayan et al., 2018; Saba et al., 2019). However, few studies had tested their usefulness in the clinical trials, and the complexity of experimental operations with lower microRNAs detection sensitivity and specificity limited its clinical application. The alteration of GM composition involves many diseases, including neuropsychiatric diseases. However, the GM composition of PSCI is still largely unknown. This study showed that the characteristic GM could be used as a diagnostic biomarker for PSCI. further, combining other valuable biomarkers is also needed to improve the accuracy of PSCI diagnosis.

Several limitations of this study should be mentioned. Multiple variables influence GM composition, and it is difficult to achieve complete standardization for all patients. Meanwhile, patients enrolled in our study tended to have lower NIHSS scores, and we did not distinguish post-stroke dementia patients from PSCI non-dementia patients, which limited the representativeness of the study. The application on the outcome of the MoCA was somewhat overemphasized. In future studies, we will use more clinical scales such as Hastgawa Dementia Scale and Wechsler Memory Scale to verify our results. Moreover, we did not investigate the GM of these patients before cognitive decline and a healthy control group without stroke, as well as the long-term follow-up, which resulted in lacking the dynamic observation of the disease. Besides, our study was a single-center study in which the number of patients was still not enough. Thus, the conclusion that GM is closely associated with PSCI may not be made quickly. Age is a vital factor contributing to GM composition, and additional experiments with larger samples in age-matched groups for the PSCI and PSNCI are needed to verify the present results.

Despite these limitations, the study has some important strengths. First, this is one of the first studies characterizing the GM in patients with PSCI, filling the GM information gap in PSCI. Second, we also investigated the risk factors for PSCI and their associations with GM. The broader connections were established between GM and the risk factors, which contributed to a better understanding of GM’s role in PSCI. Third, this study gave new clues to explore the novel diagnostic biomarkers and interventions for PSCI.

In summary, our study assessed the GM composition of PSCI patients and further indicated that the characteristic GM, especially *Enterobacteriaceae*, might facilitate the diagnosis of PSCI.

## Data Availability Statement

The datasets generated for this study can be found in the NCBI Trace Archive NCBI Sequence Read Archive, SRA accession: PRJNA588869, Temporary Submission ID: SUB6532710, the SRA records will be accessible with the following link after the indicated release date: https://www.ncbi.nlm.nih.gov/sra/PRJNA588869, moreover, we have already uploaded the clinical data and 16s data to the additional files.

## Ethics Statement

The studies involving human participants were reviewed and approved by The Ethics Committee of the Second Affiliated Hospital of Wenzhou Medical University. The patients/participants provided their written informed consent to participate in this study. Written informed consent was obtained from the individual(s) for the publication of any potentially identifiable images or data included in this article.

## Author Contributions

JS and JL conceived and designed the experiments. YL, TG, JZ, QG, XG, and XW performed the experiments and conducted the statistical analyses. All authors contributed to the article and approved the submitted version.

## Conflict of Interest

The authors declare that the research was conducted in the absence of any commercial or financial relationships that could be construed as a potential conflict of interest.
