# Supplementary material for: Gut Microbiome Signatures Are Biomarkers for Cognitive Impairment in Patients With Ischemic Stroke
Source: Front Aging Neurosci. 2020 Oct 23;12:511562. doi: 10.3389/fnagi.2020.511562 (PMC7645221; doi:10.3389/fnagi.2020.511562)
Supplement: Supplementary file 1 [file Table_1.docx]

| **Supplemental Table S1 \|** The significant differences of demographic and clinical parameters between age-matched PSCI and PSNCI groups. | | | | | | | |
| --- | --- | --- | --- | --- | --- | --- | --- |
| Characteristic |  | Age matched PSCI group  (n = 29) | |  | PSNCI group  (n = 40) |  | *P* Value |
| Male |  | 65.5 |  | | 67.5 |  | 0.863 |
| Age, years |  | 68.7 ± 10.2 |  | | 66.0 ± 10.8 |  | 0.286 |
| Divorce rate |  | 20.7 |  | | 10.0 |  | 0.369 |
| Low education (< high school) |  | 69.0 |  | | 57.5 |  | 0.332 |
| Physical activity (≥ 90 min/week) |  | 44.8 |  | | 62.5 |  | 0.145 |
| Sleep deprivation (＜ 6h) |  | 37.9 |  | | 45.0 |  | 0.557 |
| NIHSS score |  | 3 (1-5) |  | | 1 (1-2) |  | 0.001 |
| MOCA score |  | 13.5 ± 7.8 |  | | 22.3 ± 4.2 |  | < 0.001 |
| Visuospatial/executive function |  | 2 (1-3) |  | | 3 (2-3) |  | 0.001 |
| Naming |  | 1 (0-2) |  | | 2 (1-3) |  | 0.001 |
| Attention |  | 4 (1-5.5) |  | | 6 (5-6) |  | < 0.001 |
| Language |  | 2 (1-2) |  | | 3 (2-3) |  | < 0.001 |
| Abstraction |  | 0 (0-0.5) |  | | 1 (0-1) |  | 0.006 |
| Delayed recall |  | 1 (0-3) |  | | 4 (3-4) |  | < 0.001 |
| Orientation |  | 4 (2-5) |  | | 6 (5-6) |  | < 0.001 |
| Diabetes mellitus |  | 34.5 |  | | 32.5 |  | 0.863 |
| Hypertension |  | 75.9 |  | | 65.0 |  | 0.333 |
| Dyslipidemia |  | 50.0 |  | | 42.5 |  | 0.541 |
| Atrial fibrillation |  | 17.2 |  | | 7.5 |  | 0.386 |
| Stroke recurrence |  | 62.1 |  | | 20.0 |  | < 0.001 |
| BMI (kg/m^2^) |  | 25.2 ± 3.6 |  | | 25.5 ± 3.3 |  | 0.689 |
| Current smokers |  | 13.8 |  | | 15.0 |  | 1.000 |
| Alcohol drinker |  | 44.8 |  | | 43.6 |  | 0.919 |
| Hcy (umol/L) |  | 13.1 ± 5.5 |  | | 10.2 ± 2.4 |  | 0.011 |
| Dietary risks |  |  |  | |  |  |  |
| High fat |  | 62.1 |  | | 72.5 |  | 0.359 |
| Low in fruits |  | 51.7 |  | | 45.0 |  | 0.581 |
| Low in vegetables |  | 44.8 |  | | 45.0 |  | 0.989 |
| LA |  | 96.6 |  | | 67.5 |  | 0.003 |
| Brain atrophy |  | 62.1 |  | | 22.5 |  | 0.001 |
|  | | | | | | | |
