# Supplementary material for: Gut Microbiome Signatures Are Biomarkers for Cognitive Impairment in Patients With Ischemic Stroke
Source: Front Aging Neurosci. 2020 Oct 23;12:511562. doi: 10.3389/fnagi.2020.511562 (PMC7645221; doi:10.3389/fnagi.2020.511562)
Supplement: Supplementary file 2 [file Table_2.docx]

| **Supplemental Table S2 Predicted KEGG functional pathways.** | | | | | |
| --- | --- | --- | --- | --- | --- |
| KEGG ortholog functional categories | | | PSCI  mean%(SD%) | PSNCI  mean%(SD%) | P value |
| Level1 | Level2 | Level3 |  |  |  |
| Genetic Information Processing | Folding, Sorting and Degradation | Chaperones and folding catalysts | 1.313 (0.145) | 1.271 (0.135) | 0.049 |
| Unclassified | Genetic Information Processing | Protein folding and associated processing | 1.154 (0.088) | 1.112 (0.082) | 0.026 |
| Unclassified | Genetic Information Processing | Transcription related proteins | 0.012 (0.010) | 0.009 (0.008) | 0.030 |
| Metabolism | Energy Metabolism | Nitrogen metabolism | 0.512 (0.077) | 0.487 (0.064) | 0.036 |
| Metabolism | Energy Metabolism | Sulfur metabolism | 0.235 (0.026) | 0.245 (0.023) | 0.017 |
| Unclassified | Metabolism | Glycan biosynthesis and metabolism | 0.081 (0.030) | 0.069 (0.020) | 0.014 |
| Unclassified | Metabolism | Nucleotide metabolism | 0.080 (0.022) | 0.072 (0.025) | 0.033 |
| Metabolism | Enzyme Families | Protein kinases | 0.259 (0.030) | 0.248 (0.025) | 0.041 |
| Metabolism | Carbohydrate Metabolism | Propanoate metabolism | 0.184 (0.016) | 0.177 (0.016) | 0.013 |
| Metabolism | Metabolism of Cofactors and Vitamins | Porphyrin and chlorophyll metabolism | 1.400 (0.229) | 1.454 (0.208) | 0.038 |
| Metabolism | Metabolism of Cofactors and Vitamins | Pantothenate and CoA biosynthesis | 0.790 (0.030) | 0.806 (0.028) | 0.002 |
| Metabolism | Metabolism of Cofactors and Vitamins | Nicotinate and nicotinamide metabolism | 0.691 (0.044) | 0.708 (0.053) | 0.037 |
| Metabolism | Metabolism of Cofactors and Vitamins | Thiamine metabolism | 0.674 (0.042) | 0.711 (0.033) | ＜0.001 |
| Metabolism | Amino Acid Metabolism | Phenylalanine, tyrosine and tryptophan biosynthesis | 1.301 (0.092) | 1.352 (0.077) | 0.004 |
| Metabolism | Amino Acid Metabolism | Arginine and proline metabolism | 1.155 (0.126) | 1.198 (0.119) | 0.038 |
| Metabolism | Amino Acid Metabolism | Histidine metabolism | 0.823 (0.141) | 0.865 (0.144) | 0.031 |
| Metabolism | Amino Acid Metabolism | Alanine, aspartate and glutamate metabolism | 0.737 (0.036) | 0.750 (0.037) | 0.04 |
| Metabolism | Amino Acid Metabolism | Valine, leucine and isoleucine biosynthesis | 0.511 (0.072) | 0.536 (0.074) | 0.016 |
| Metabolism | Amino Acid Metabolism | Valine, leucine and isoleucine degradation | 0.095 (0.013) | 0.101 (0.022) | 0.050 |
| Unclassified | Metabolism | Carbohydrate metabolism | 0.293 (0.059) | 0.312 (0.061) | 0.032 |
| Metabolism | Lipid Metabolism | Primary bile acid biosynthesis | 0.029 (0.008) | 0.033 (0.009) | 0.003 |
| Metabolism | Lipid Metabolism | Secondary bile acid biosynthesis | 0.029 (0.008) | 0.033 (0.008) | 0.005 |
| Metabolism | Lipid Metabolism | Linoleic acid metabolism | 0.014 (0.003) | 0.016 (0.003) | 0.006 |
| Abbreviations: KEGG, Kyoto Encyclopedia of Genes and Genomes; PSCI, post-stroke cognitive impairment; PSNCI, post-stroke noncognitive impairment; SD: standard deviation. | | | | | |
