# Supplementary material for: Gut Microbiome Signatures Are Biomarkers for Cognitive Impairment in Patients With Ischemic Stroke
Source: Front Aging Neurosci. 2020 Oct 23;12:511562. doi: 10.3389/fnagi.2020.511562 (PMC7645221; doi:10.3389/fnagi.2020.511562)
Supplement: Supplementary file 3 [file Table_3.docx]

| **Supplemental Table S3 The logistic regression models of the characteristic GM and PSCI, unadjusted (model 1), adjusted for age (model 2), and adjusted for age and risk factors for PSCI (model 3)** | | | | | | | | | |
| --- | --- | --- | --- | --- | --- | --- | --- | --- | --- |
| Characteristic  bacteria | Model 1 | | | Model 2 | | | Model 3 | | |
|  | OR | 95%CI | *P* | OR | 95%CI | *P* | OR | 95%CI | *P* |
| ***Prevotella*** |  |  | 0.097 |  |  | 0.106 |  |  | 0.054 |
| T1:first tertile (reference) | 1 |  |  | 1 |  |  | 1 |  |  |
| T2:second tertile | 2.976 | 0.885-10.010 | 0.078 | 2.906 | 0.846-9.979 | 0.090 | 1.626 | 0.236-11.225 | 0.622 |
| T3:third tertile | 3.581 | 0.995-12.888 | 0.051 | 3.719 | 0.979-14.123 | 0.054 | 12.449 | 1.503-103.078 | 0.019 |
|  |  |  |  |  |  |  |  |  |  |
| ***Streptococcus*** |  |  | 0.122 |  |  | 0.092 |  |  | 0.199 |
| T1:first tertile (reference) | 1 |  |  | 1 |  |  | 1 |  |  |
| T2:second tertile | 1.742 | 0.311-9.749 | 0.528 | 1.779 | 0.299-10.568 | 0.526 | 0.464 | 0.023-9.275 | 0.615 |
| T3:third tertile | 7.656 | 0.922-63.580 | 0.059 | 9.063 | 1.012-81.153 | 0.049 | 6.503 | 0.253-167.245 | 0.258 |
|  |  |  |  |  |  |  |  |  |  |
| ***Klebsiella*** |  |  | 0.115 |  |  | 0.198 |  |  | 0.218 |
| T1:first tertile (reference) | 1 |  |  | 1 |  |  | 1 |  |  |
| T2:second tertile | 3.764 | 1.076-13.171 | 0.038 | 3.286 | 0.898-12.029 | 0.072 | 6.331 | 0.593-67.637 | 0.127 |
| T3:third tertile | 1.521 | 0.430-5.377 | 0.515 | 1.524 | 0.415-5.590 | 0.526 | 0.958 | 0.117-7.873 | 0.968 |
|  |  |  |  |  |  |  |  |  |  |
| ***Enterobacteriaceae*** |  |  | 0.116 |  |  | 0.169 |  |  | 0.035 |
| T1:first tertile (reference) | 1 |  |  | 1 |  |  | 1 |  |  |
| T2:second tertile | 1.358 | 0.357-5.167 | 0.654 | 1.273 | 0.317-5.113 | 0.734 | 3.680 | 0.439-30.836 | 0.230 |
| T3:third tertile | 3.978 | 0.979-16.160 | 0.054 | 3.503 | 0.834-14.725 | 0.087 | 59.721 | 2.677-1332.158 | 0.010 |
|  |  |  |  |  |  |  |  |  |  |
| ***Lactobacillales*** |  |  | 0.474 |  |  | 0.364 |  |  | 0.666 |
| T1:first tertile (reference) | 1 |  |  | 1 |  |  | 1 |  |  |
| T2:second tertile | 1.183 | 0.207-6.746 | 0.850 | 0.909 | 0.149-5.547 | 0.918 | 2.541 | 0.100-64.853 | 0.573 |
| T3:third tertile | 0.441 | 0.057-3.426 | 0.433 | 0.300 | 0.035-2.560 | 0.271 | 0.968 | 0.027-34.326 | 0.986 |
| Note: We divided the abundance of GM into trisection and used the first third as the reference category indicator. model 1, unadjusted; model 2, adjusted for age; model 3, adjusted for age, NIHSS score, stroke recurrence, Hcy, LA and brain atrophy. GM, gut microbiota; PSCI, post-stroke cognitive impairment; OR, odds ratio; 95% CI, 95% confidence interval. | | | | | | | | | |
