# Supplementary figures and images for: Gut Microbiome Signatures Are Biomarkers for Cognitive Impairment in Patients With Ischemic Stroke
Source: Front Aging Neurosci. 2020 Oct 23;12:511562. doi: 10.3389/fnagi.2020.511562 (PMC7645221; doi:10.3389/fnagi.2020.511562)

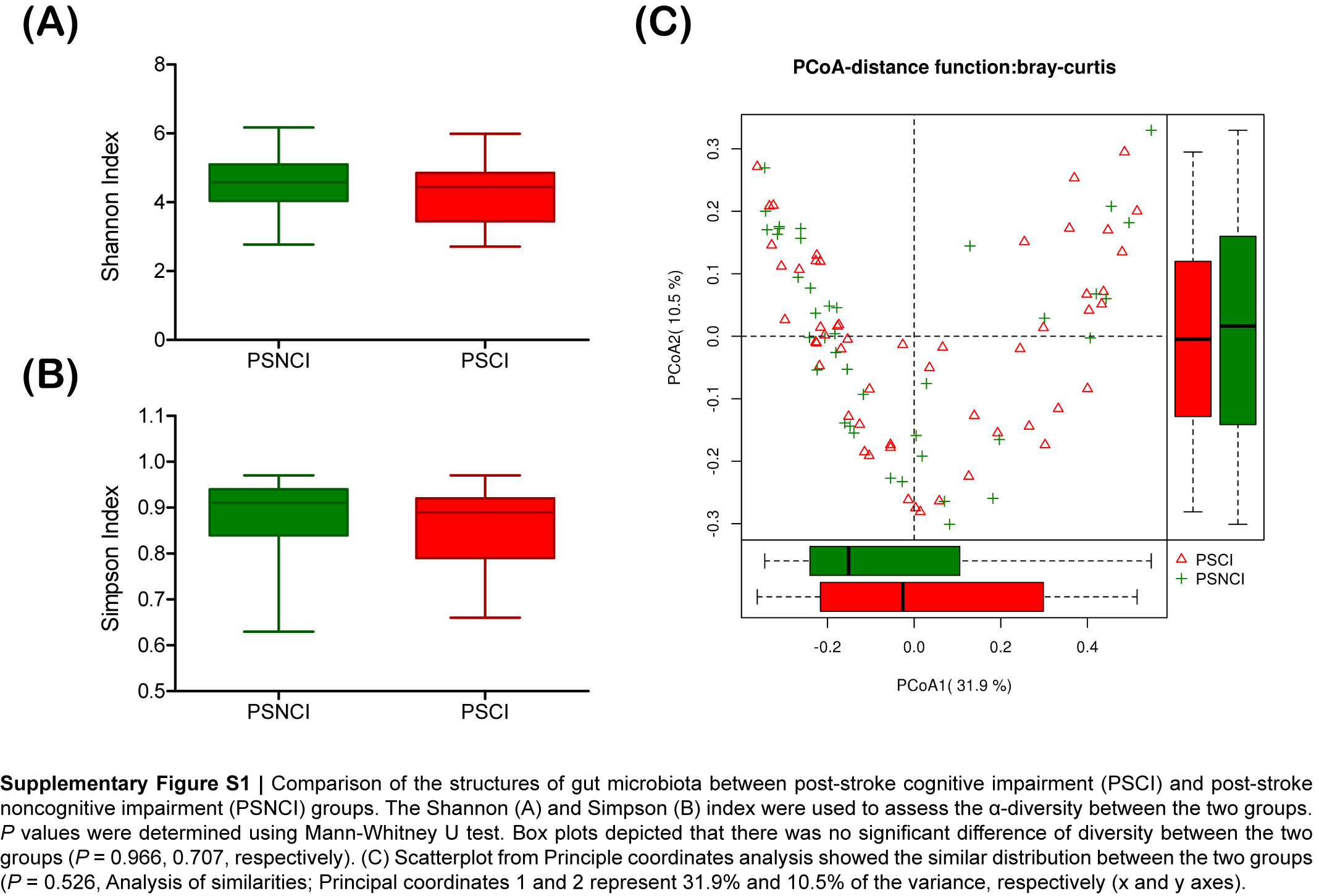

Supplement: Supplementary file 7 [file Image_1.TIF]

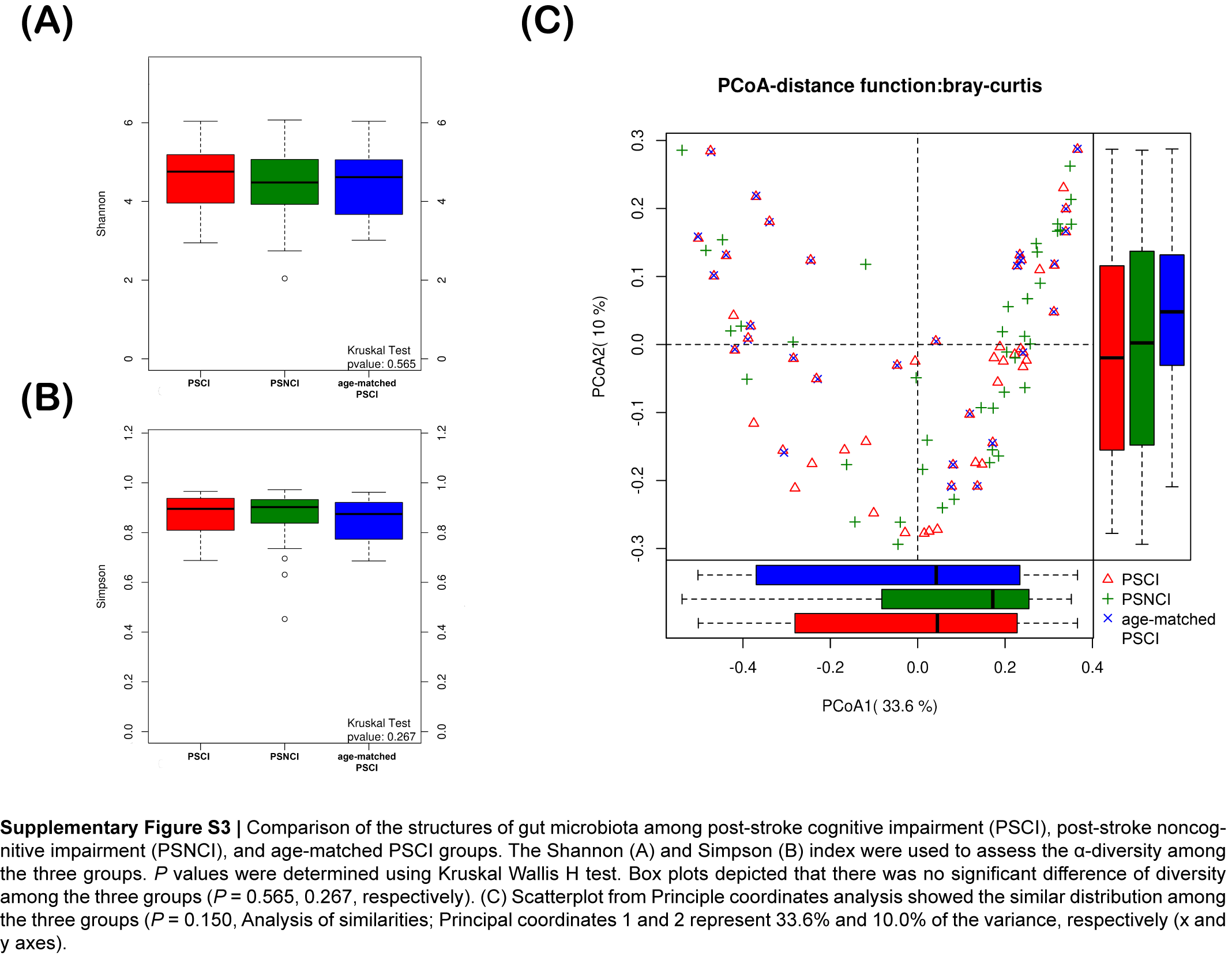

Supplement: Supplementary file 8 [file Image_2.TIF]

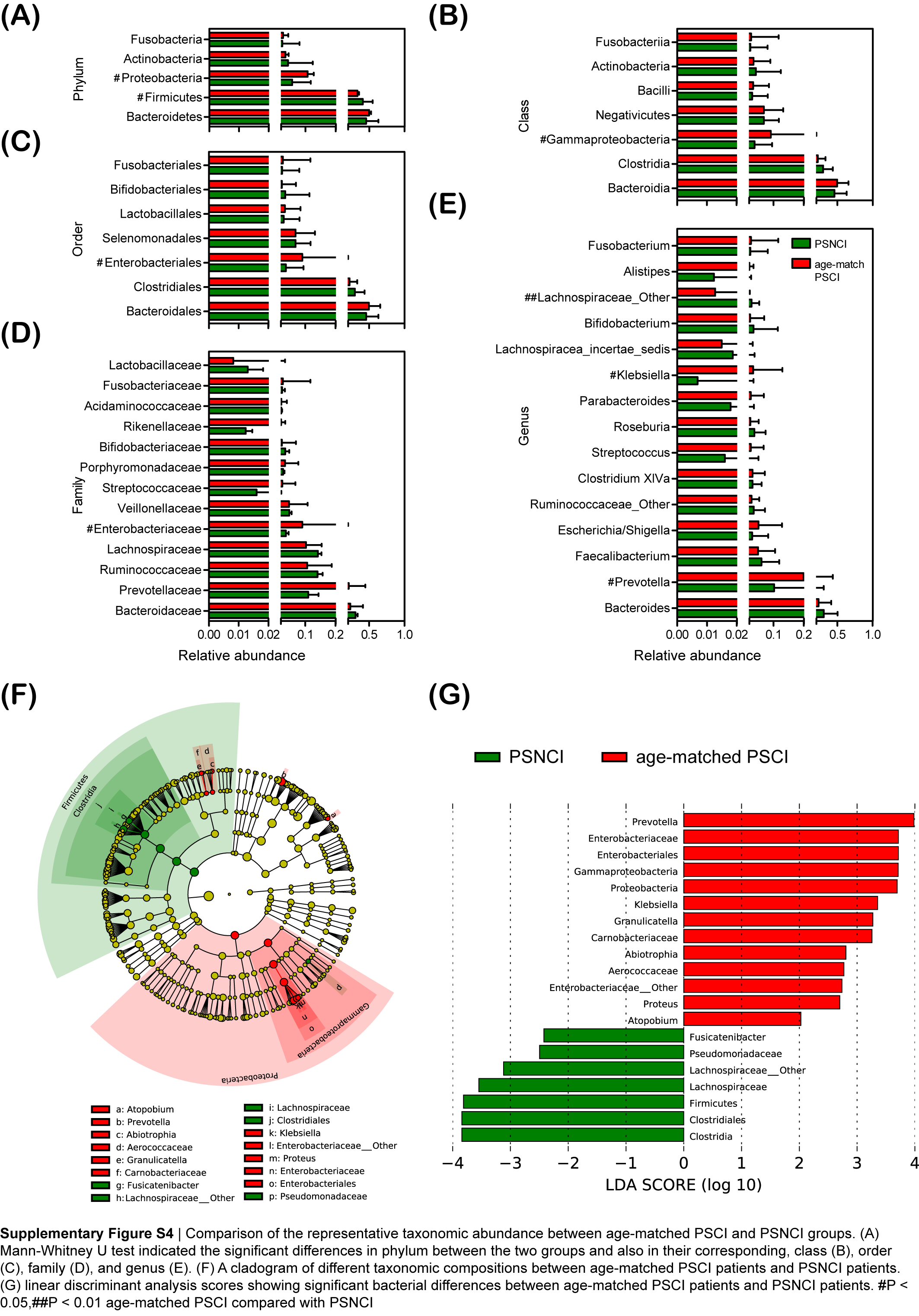

Supplement: Supplementary file 9 [file Image_3.TIF]

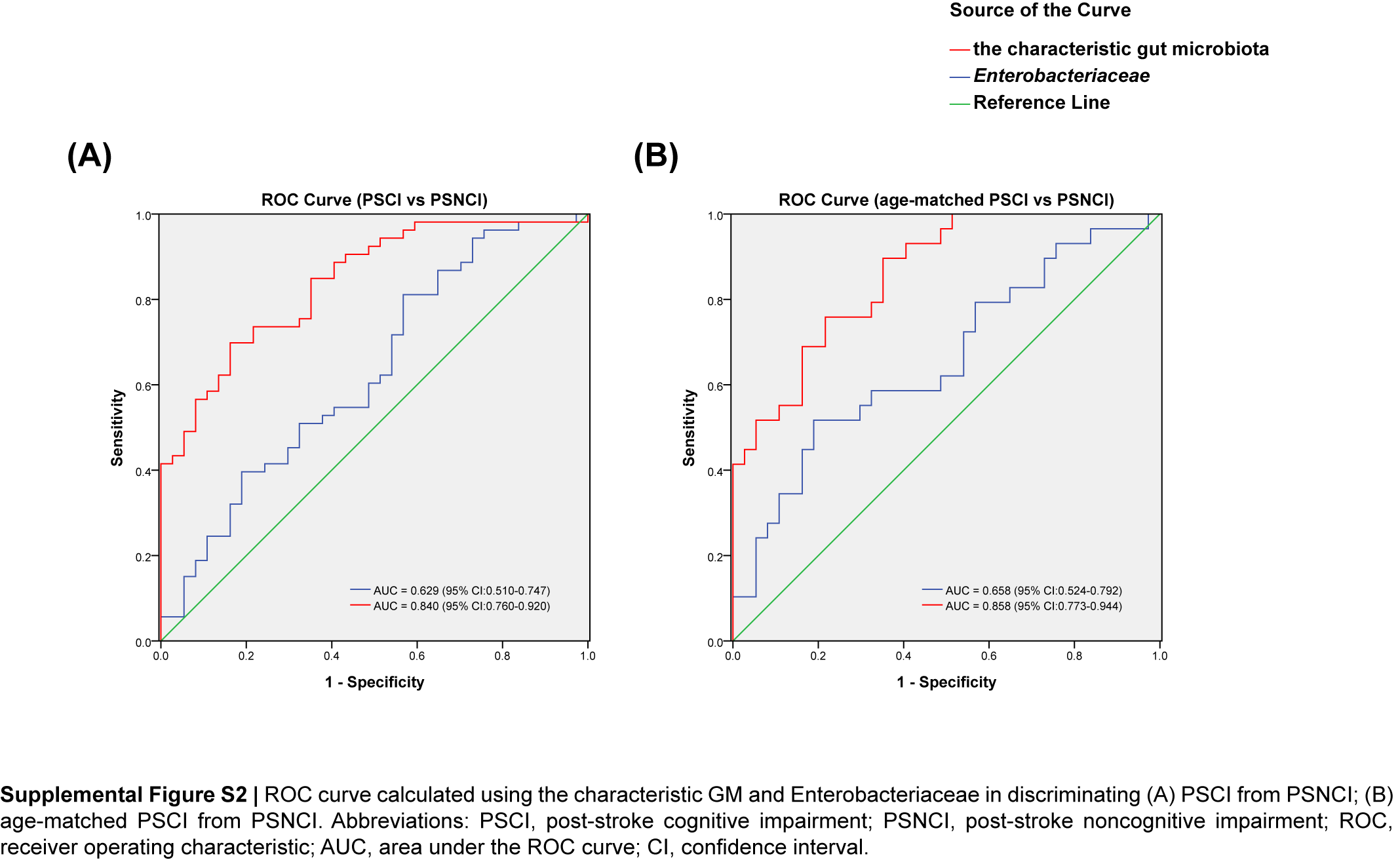

Supplement: Supplementary file 10 [file Image_4.TIF]

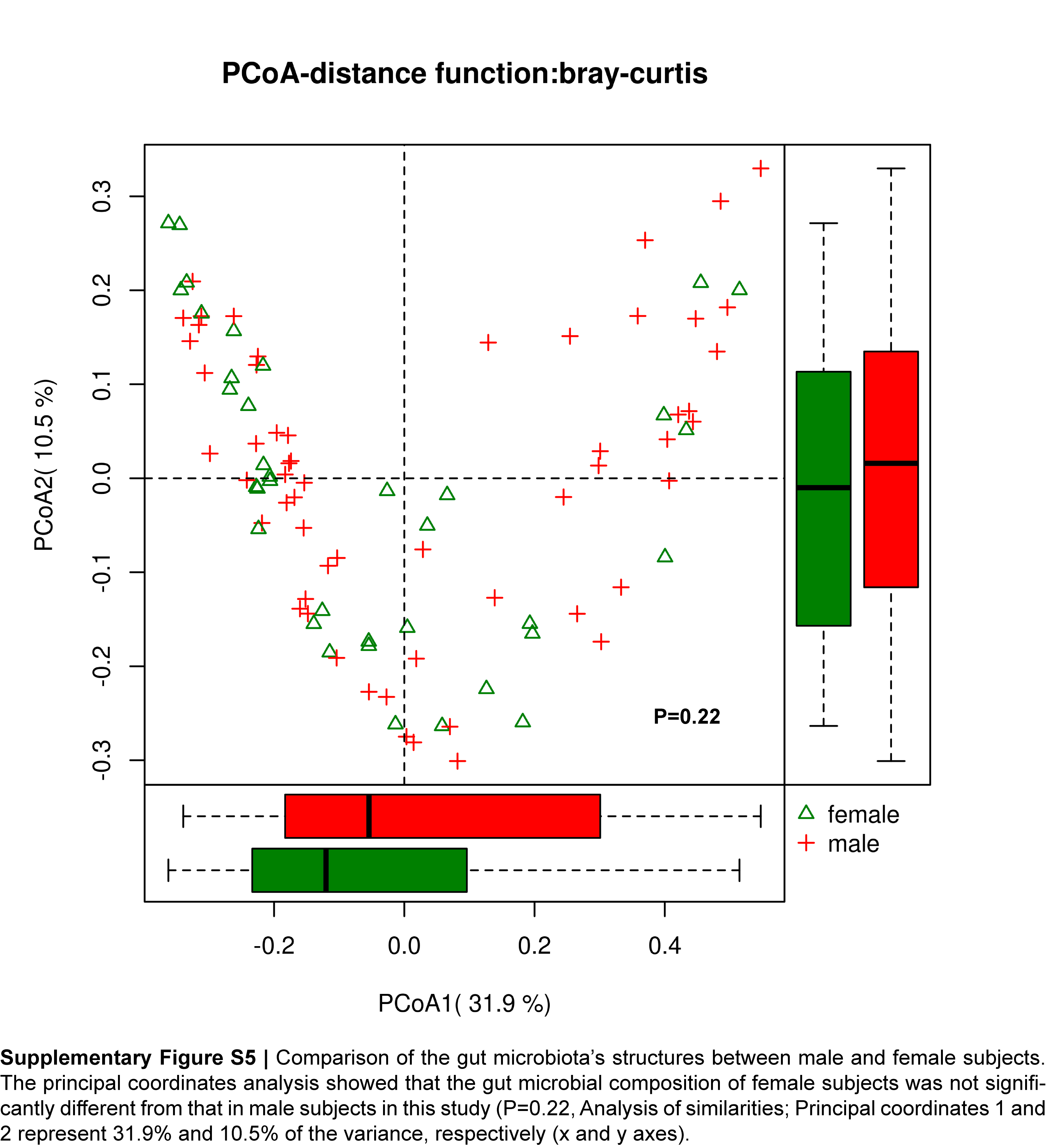

Supplement: Supplementary file 11 [file Image_5.TIF]

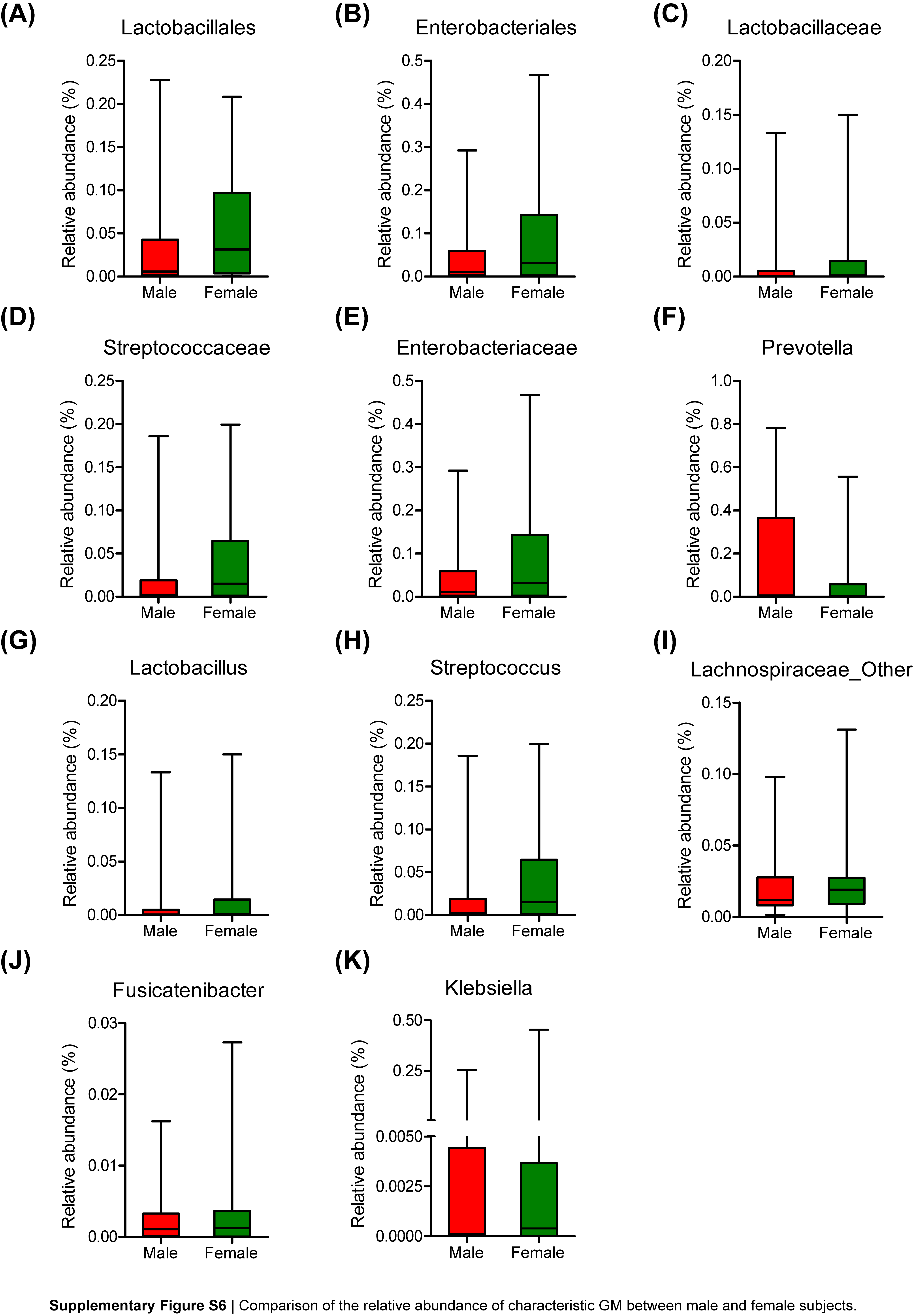

Supplement: Supplementary file 12 [file Image_6.TIF]
